# Pharmacovigilance analysis of iodinated contrast media related respiratory adverse effects based on the FDA adverse event reporting system

**DOI:** 10.3389/fphar.2026.1737135

**Published:** 2026-03-16

**Authors:** Yang Rui, Tianyuan Xin, Yu Chen, Beiyi Xiang, Changwen Chen, Lin Zhang, Zhe Chen, Zhigang Qian

**Affiliations:** 1 Laboratory of Cough, Affiliated Kunshan Hospital of Jiangsu University, Kunshan Key Laboratory of Chronic Cough, Suzhou, China; 2 Department of Oncology, Changshu Hospital Affiliated to Soochow University, Changshu No.1 People’s Hospital, Suzhou, China; 3 Department of Emergency, the Third People’s Hospital of Yunnan Province, Kunming, China

**Keywords:** FAERS, iodinated contrast media, pharmacovigilance, respiratory adverse events, signal detection

## Abstract

**Aim:**

Iodinated contrast media (ICM) serve as a cornerstone in diagnostic imaging; however, the risk profile of their respiratory system adverse reactions remains inadequately characterized. This study aims to quantify and compare the respiratory system adverse reaction signals associated with four widely utilized non-ionic ICMs—iohexol, iopamidol, iopromide, and ioversol—using the United States Food and Drug Administration’s Adverse Event Reporting System (FAERS) database.

**Methods:**

We extracted data from the FAERS database spanning the first quarter of 2004 to the fourth quarter of 2024. Signal detection was performed employing the Reporting Odds Ratio (ROR), Proportional Reporting Ratio (PRR), Bayesian Confidence Propagation Neural Network (BCPNN), and Multi-Item Gamma Poisson Shrinker (MGPS) methodologies to identify statistically significant drug-event associations.

**Results:**

A total of 9,682 adverse event (AE) reports related to the respiratory system were analyzed, encompassing 53 distinct respiratory-related events. All four ICMs demonstrated a significant correlation with respiratory diseases. Notably, the analysis revealed that certain adverse reactions were not covered in the prescribing information. Moreover, sneezing and laryngeal edema exhibited unusually high signal intensities across all four ICMs.

**Conclusion:**

This study reveals heterogeneity in the respiratory system risk profiles among the four commonly used non-ionic ICMs. These findings suggest that clinical decision-making should incorporate the distinct risk profiles of specific ICMs, and individualized monitoring strategies should be implemented for high-risk patients.

## Introduction

Iodinated contrast media (ICM) serve as the cornerstone of modern medical imaging, widely utilized in examinations such as enhanced computed tomography (CT), angiography, and urography. However, with the extensive application of ICM in clinical practice, the challenges associated with the prevention and management of their related adverse events (AEs) are becoming increasingly prominent. For instance, issues such as delayed hypersensitivity reactions, acute kidney injury, and thyroid dysfunction are associated with ICM (Torres et al., 2021; Davenport, 2023; Inoue et al., 2023). Currently, ICM employed in clinical settings are primarily non-ionic agents, including iohexol and iopamidol. Non-ionic ICM exhibit a higher safety profile compared to ionic preparations, attributed to their lower osmotic pressure characteristics (Zeng et al., 2024). Previous research on the AEs of ICM has predominantly focused on allergic reactions, encompassing their diagnosis, treatment, and potential complications, such as the induction of dermatitis herpetiformis (Kang et al., 2022; Umakoshi et al., 2022; Tsutsui et al., 2022). Despite the widespread recognition of the overall safety of non-ionic ICM, the potential for specific safety risk differences among various formulations remains uncertain. Large-scale reviews have identified the respiratory system as a common target organ for acute adverse reactions associated with non-ionic ICM (Suh et al., 2019). Preliminary investigations utilizing spontaneous reporting systems have suggested heterogeneity in the AE profiles across different ICMs. However, these analyses have not specifically focused on respiratory events, nor have they conducted direct comparative assessments of the most commonly used agents, such as iohexol and iopamidol (Tan et al., 2025; Zhang et al., 2025). Consequently, a systematic, quantitative comparison of respiratory system risk signals among frontline clinical non-ionic ICMs is currently lacking.

The U.S. Food and Drug Administration Adverse Event Reporting System (FAERS), as a spontaneous drug safety surveillance database primarily focused on the U.S. population, has amassed over 20 million AE reports, providing a unique resource for identifying rare, serious, or emerging drug risks (Zhou and Hultgren, 2020). Its primary advantage lies in its capacity to capture AE characteristics across diverse populations, comorbidities, and medication patterns in real-world settings. This makes it particularly suitable for comparing risk differences among similar drugs. However, a gap remains in the current literature regarding the respiratory AEs associated with ICM within FAERS. To address this gap, the present study utilizes FAERS data from 2004 to 2024. Through multidimensional pharmacovigilance signal mining and analysis, we systematically compare the AE signal intensity and clinical phenotype differences of four types of ICM—iohexol, iopamidol, ioversol and iopromide—specifically in relation to the respiratory system.

## Methods

### Source of data

The FAERS database is an open-access pharmacovigilance repository that contains millions of AE reports, primarily submitted by healthcare professionals, employees of pharmaceutical companies, and various stakeholders (Marwitz et al., 2020). We employed the generic and brand names of four common ICMs as keywords to retrieve all AE reports related to these agents. The time frame for this study’s search spans from the first quarter of 2004 to the fourth quarter of 2024. The examined ICMs include “Iohexol/Omnipaque,” “Iopamidol/Isovue/Niopam,” “Iopromide/Ultravist,” and “Ioversol/Optiray.” This study primarily focuses on four types of non-ionic ICM due to their high clinical usage rates in the U.S. market and the substantial number of related reports in the FAERS database. Other non-ionic ICM, such as iomeprol and iobitridol, have not yet received FDA approval. Iodixanol is primarily indicated for specific clinical scenarios, such as cardiovascular angiography, and its iso-osmolar physiologic properties differ from the low-osmolar ICMs covered in this study. Moreover, preliminary data screening revealed a negligible number of respiratory-related AE reports for iodixanol in the FAERS database. Consequently, considering its clinical applicability, mechanism of action, and data sufficiency, iodixanol was excluded from the present analysis. In this study, we evaluated various parameters related to respiratory, thoracic, and mediastinal disorders. The data sources primarily include demographic information (DEMO), drug usage records (DRUG), AE records (REAC), patient outcomes (OUTC), drug treatment duration (THER), drug indications (INDI), and sources of AEs (RPSR) (Sakaeda et al., 2013).

### Data processing

Due to the nature of the FAERS as a spontaneous reporting system, instances of duplicate reports may occur. In this study, we conducted deduplication of the original data through the following steps: we selected the fields PRIMARYID, CASEID, and FDA_DT from the DEMO table, sorting them by CASEID, FDA_DT, and PRIMARYID. When processing reports with identical CASEIDs, we prioritize retaining the report with the highest FDA_DT value. If both CASEID and FDA_DT are identical, we select the report with the largest PRIMARYID value for retention. The FAERS database uses the ROLE_CD field to denote the suspect role of a drug in an AE, which primarily includes: PS (Primary Suspect): the drug primarily suspected to cause the event; SS (Secondary Suspect): the drug secondarily suspected; C (Concomitant): a co-administered drug; I (Interacting): a drug involved in an interaction. To maximize the certainty of the association between AEs and the target ICM, the primary analysis in this study only included reports where the target drug was designated as “PS.” In cases where multiple target agents were concurrently marked as “PS” within the same report (e.g., both iohexol and iopamidol were labeled as PS), the case was counted for each agent. However, during the aggregation of total case numbers, duplicate entries were removed based on patient identifiers and the date of event occurrence, ensuring that each patient was counted only once per event. In addition, some problematic data will be removed, primarily those adverse reactions that are unrelated to medication, such as product issues, medical procedures, and social environmental factors. Subsequently, we applied the latest version of the MedDRA dictionary (MedDRA27.1) to encode the AE names in the FAERS database with preferred term (PT) and corresponding system organ class (SOC) codes (Tieu and Breder, 2018). Finally, we filtered out records in the adverse drug reaction information table that pertain to respiratory, thoracic, and mediastinal disorders for data analysis.

### Statistical analysis

In this study, to enhance the accuracy of signal detection, we primarily employed four data mining algorithms—ROR, PRR, BCPNN, and MGPS. All four algorithms mentioned above are based on the fourfold table of proportional imbalance method (Supplementary Table S1) and utilize the corresponding formulas to calculate the signals of ICM (Supplementary Table S2). The thresholds for defining a positive signal for each algorithm were set as follows. ROR: A signal is considered potential if the case count (a) is ≥ 3 and the lower bound of the 95% confidence interval (CI) for the ROR exceeds 1 (van Puijenbroek et al., 2002). PRR: A signal is considered potential if the case count (a) is ≥ 3, the PRR is ≥ 2, and the chi-square (χ^2^) statistic is ≥ 4 (Evans et al., 2001). BCPNN: A signal is considered potential if the lower bound of the 95% CI for the Information Component (IC) is greater than 0 (Bate, 2007). MGPS: A signal is considered potential if the lower bound of the 95% CI for the Empirical Bayes Geometric Mean (EBGM) is greater than 2 (Trippe et al., 2017). To maximize specificity (i.e., minimize the risk of false positives), this study strictly defined a “valid signal” as a drug-event pair that simultaneously meets the positive thresholds of all four algorithms (Sakaeda et al., 2013). While this approach may reduce sensitivity to some extent, it provides a more reliable method for screening robust drug safety signals—especially in spontaneous reporting databases like FAERS that are prone to reporting bias—thereby offering results that are more valuable for clinical decision-making. All data processing and statistical analyses were performed in R (version 4.4.2) using packages such as “data.table,” “dplyr,” “ggplot2,” and “PhViD.”

## Results

**FIGURE 1:**
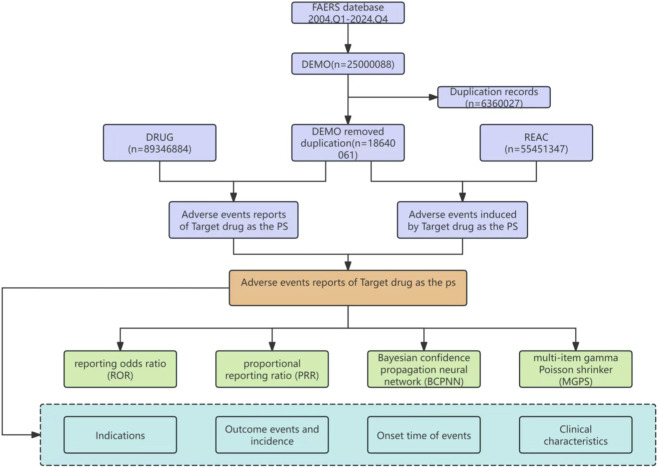
Flowchart of the FAERS database’s pipeline for screening ICM-associated respiratory AEs. Illustrates the data cleaning and analysis process employed in this study. By eliminating duplicate and problematic data and utilizing four statistical methods, the research investigated the common AEs of ICM within the respiratory system.

In this study, we conducted a comprehensive search of the FAERS database, covering 80 quarters from the first quarter of 2004 to the fourth quarter of 2024, which yielded a total of 25,000,088 entries. After removing duplicates and problematic data, we included 18,640,061 complete data reports. We performed an extensive search for all keywords related to ICM, resulting in the collection of 55,451,347 adverse reaction records across all systems. Within the context of respiratory, thoracic, and mediastinal disorders, we compiled the following statistics on related AE reports: iohexol (3,095 cases), iopamidol (855 cases), iopromide (3,824 cases) and ioversol (1,908 cases). The specific data cleaning and analysis process is illustrated in Figure 1.

### Patients characteristics

**FIGURE 2:**
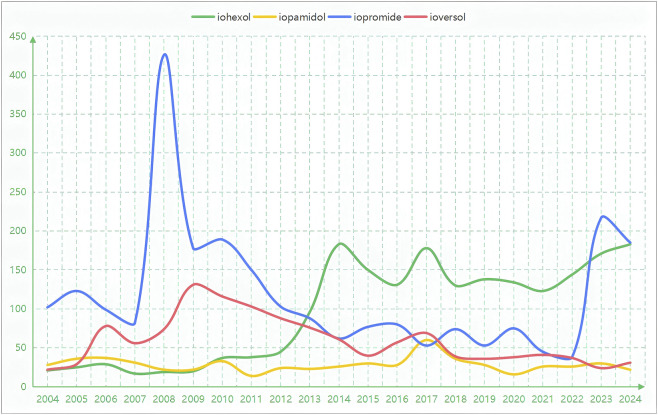
Annual distribution of reported AEs in the respiratory system for four ICM drugs. Illustrates the annual quantity of respiratory system-related AEs associated with the target drug in this study. Notably, iohexol and iopromide exhibit significant trends in the reported numbers over the years.

A total of 6,355 patients experienced AEs related to the respiratory system with four ICMs, including 2,012 cases with iohexol, 598 cases with iopamidol, 2,499 cases with iopromide, and 1,246 cases with ioversol. The basic information on AE reports for the four drugs in the respiratory system is presented in Table 1. In the respiratory AE reports for these agents, gender distribution was relatively consistent, with a higher prevalence of female patients. After excluding missing values, the number of female patients (3,493 cases, 63.47%) was significantly higher than that of male patients (2,010 cases, 36.53%). Age distribution trends were largely similar, with the 45–65 age group being the most prevalent and patients under the age of 18 being the least represented. The primary reporting country for the four drugs was predominantly the United States. Given the significant differences in reporting countries for the four drugs, except for the country with the highest number of reports, they are no longer listed separately in Table 1. The majority of reporters were pharmacists, followed by physicians and consumers. The onset time trends of respiratory AEs induced by the four drugs after administration were roughly similar, primarily concentrated within 0–30 days post-administration. Among them, iohexol accounted for the highest proportion at 49.17%, while the other three drugs exhibited roughly equivalent proportions, all around 40%. Additionally, we also calculated the median time to onset for four different drugs concerning each significant signal. For example, the median time to the onset of dyspnoea when using iohexol was 1 day, while for iopromide, it was 5.5 days. The detailed median times of occurrence are available in Supplementary Table S3. The most common outcome of the illness was hospitalization, followed by life-threatening conditions and death, with a very small proportion of individuals experiencing disability. Regarding the annual distribution of the four drugs, iohexol has demonstrated a significant growth trend since 2014, while iopromide exhibited two distinct peaks in 2008 and 2023, respectively (Figure 2).

**TABLE 1:** Basic information reported on AE in the respiratory system for four ICM drugs.

Item	Iohexol	Iopamidol	Iopromide	Ioversol
Gender n (%)				
Female	952 (47.32)	390 (65.22)	1,455 (58.22)	696 (55.86)
Male	564 (28.03)	199 (33.28)	884 (35.37)	363 (29.13)
Unknow	496 (24.65)	9 (1.51)	160 (6.4)	187 (15.01)
Age n (%)				
<18	49 (2.44)	10 (1.67)	52 (2.08)	49 (3.93)
18–44	426 (21.17)	128 (21.4)	628 (25.13)	274 (21.99)
45–64	539 (26.79)	211 (35.28)	865 (34.61)	336 (26.97)
65–75	239 (11.88)	84 (14.05)	396 (15.85)	154 (12.36)
>75	161 (8)	85 (14.21)	233 (9.32)	111 (8.91)
Unknow	598 (29.72)	80 (13.38)	325 (13.01)	322 (25.84)
Weight n (%) (kg)				
<73	322 (16)	107 (17.89)	481 (19.25)	162 (13)
73–87	150 (7.46)	42 (7.02)	232 (9.28)	92 (7.38)
88–104	90 (4.47)	40 (6.69)	135 (5.4)	38 (3.05)
>104	82 (4.08)	32 (5.35)	63 (2.52)	21 (1.69)
Unknow	1,368 (67.99)	377 (63.04)	1,588 (63.55)	933 (74.88)
Reporter n (%)				
Pharmacist	797 (39.61)	182 (30.44)	508 (20.33)	233 (18.7)
Physician	296 (14.71)	65 (10.87)	399 (15.97)	172 (13.8)
Consumer	67 (3.33)	52 (8.7)	292 (11.69)	63 (5.06)
Lawyer	1 (0.05)	1 (0.17)	2 (0.08)	1 (0.08)
Other	784 (38.97)	229 (38.29)	1,260 (50.42)	756 (60.67)
Unknown	67 (3.33)	69 (11.54)	38 (1.52)	21 (1.69)
Onset time n (%) (day)				
<30	826 (49.17)	202 (40)	864 (39.49)	467 (40.43)
30–180	687 (40.89)	140 (27.72)	760 (34.73)	415 (35.93)
180–360	93 (5.54)	111 (21.98)	268 (12.25)	200 (17.32)
>720	41 (2.44)	41 (8.12)	251 (11.47)	48 (4.16)
360–720	33 (1.96)	11 (2.18)	45 (2.06)	25 (2.16)
Outcome (%)				
Hospitalization	550 (23.74)	200 (26.39)	644 (20.71)	317 (21.94)
Life-threatening	324 (13.98)	103 (13.59)	522 (16.79)	211 (14.6)
Death	144 (6.22)	84 (11.08)	178 (5.72)	62 (4.29)
Recovered	35 (1.51)	19 (2.51)	25 (0.8)	44 (3.05)
Disability	23 (0.99)	13 (1.72)	17 (0.55)	7 (0.48)
Other	950 (41)	248 (32.72)	1,031 (33.15)	484 (33.5)
Unknown	291 (12.56)	91 (12.01)	693 (22.28)	320 (22.15)

### Signal strength analysis of respiratory AEs

The signal strengths of all four drugs were greater than 1 in the category of “respiratory, thoracic and mediastinal disorders”: iohexol (ROR: 3.90, 95% CI: 3.75–4.05), iopamidol (ROR: 2.75, 95% CI: 2.56–2.96), iopromide (ROR: 3.97, 95% CI: 3.85–4.13), and ioversol (ROR: 5.07, 95% CI: 4.83–5.34).

### Disproportionality analysis of AEs for four ICM drugs

There were a total of 36 AEs of iohexol that met the positive criteria for the four methods (Table 2). The most frequently reported AEs associated with iohexol were primarily dyspnoea and sneezing, with over 500 cases documented. The strongest signals were noted for sneezing, laryngeal obstruction, and nasal pruritus. Additionally, we identified AE signals for sneezing (ROR: 80.98, 95% CI: 74.17–88.42), yawning (ROR: 7.11, 95% CI: 3.19–15.85), respiratory acidosis (ROR: 4.95, 95% CI: 2.22–11.03), and dysphonia (ROR: 4.89, 95% CI: 3.97–6.03), none of which are included in the iohexol drug label.

**TABLE 2:** Positive signal for iohexol-related respiratory AEs.

Sort	PT	N	ROR (95%CI)	PRR (X^2^)	IC025	EBGM05
1	Dyspnoea	686	4.01 (3.72,4.33)	3.91 (1,494.83)	1.85	3.62
2	Sneezing	525	80.98 (74.17,88.42)	78.79 (39,266.64)	5.94	70.27
3	Cough	266	3.15 (2.79,3.55)	3.12 (383.68)	1.45	2.76
4	Throat irritation	215	16.03 (14.01,18.35)	15.86 (2,980.07)	3.69	13.79
5	Throat tightness	191	23.18 (20.09,26.75)	22.96 (3,982.11)	4.14	19.75
6	Nasal congestion	107	6.11 (5.05,7.39)	6.08 (453.99)	2.26	5.02
7	Dysphonia	88	4.89 (3.97,6.03)	4.87 (270.8)	1.92	3.95
8	Wheezing	73	4.24 (3.37,5.34)	4.23 (179.94)	1.68	3.36
9	Pharyngeal oedema	58	11.06 (8.54,14.32)	11.03 (527.25)	2.86	8.49
10	Respiratory distress	55	6.37 (4.89,8.3)	6.36 (247.75)	2.15	4.87
11	Respiratory arrest	50	5.53 (4.19,7.31)	5.52 (184.93)	1.94	4.18
12	Tachypnoea	49	11.86 (8.96,15.71)	11.84 (484.23)	2.87	8.90
13	Laryngeal oedema	48	25.09 (18.87,33.34)	25.03 (1,097.73)	3.65	18.67
14	Bronchospasm	44	9.73 (7.24,13.09)	9.71 (342.79)	2.59	7.20
15	Pharyngeal swelling	38	14.33 (10.41,19.71)	14.3 (467.86)	2.95	10.35
16	Oropharyngeal discomfort	24	9.44 (6.32,14.09)	9.43 (180.19)	2.24	6.29
17	Stridor	19	20.76 (13.22,32.6)	20.74 (354.44)	2.73	13.11
18	Apnoea	18	7.19 (4.53,11.42)	7.18 (95.56)	1.77	4.51
19	dry throat	18	5.75 (3.62,9.13)	5.75 (70.42)	1.54	3.61
20	Obstructive airways disorder	16	4.84 (2.96,7.91)	4.84 (48.64)	1.28	2.96
21	Nasal pruritus	15	41.17 (24.72,68.54)	41.13 (579.16)	2.82	24.37
22	Nasal obstruction	14	29.26 (17.28,49.54)	29.24 (378)	2.59	17.10
23	Acute pulmonary oedema	13	7.3 (4.23,12.58)	7.29 (70.44)	1.56	4.22
24	Hypopnoea	13	12.45 (7.22,21.48)	12.45 (136.27)	2.00	7.19
25	Choking sensation	10	5.89 (3.17,10.96)	5.89 (40.51)	1.15	3.16
26	Laryngeal discomfort	8	42.29 (21.04,85)	42.27 (317.73)	1.95	20.73
27	Throat clearing	7	6.53 (3.11,13.71)	6.53 (32.7)	0.93	3.10
28	Respiratory tract oedema	7	32.86 (15.6,69.22)	32.85 (213.71)	1.69	15.42
29	Laryngospasm	7	8.07 (3.84,16.95)	8.07 (43.24)	1.08	3.83
30	Pharyngeal paraesthesia	6	13.88 (6.22,30.95)	13.87 (71.34)	1.19	6.19
31	Respiratory acidosis	6	4.95 (2.22,11.03)	4.95 (18.88)	0.57	2.22
32	Yawning	6	7.11 (3.19,15.85)	7.11 (31.43)	0.83	3.18
33	Larynx irritation	4	56.41 (20.97,151.75)	56.4 (213.5)	0.91	20.57
34	Laryngeal obstruction	4	76.08 (28.19,205.34)	76.06 (288.71)	0.93	27.47
35	Laryngeal pain	3	25.81 (8.28,80.44)	25.81 (70.91)	0.39	8.21
36	Rhonchi	3	6.83 (2.2,21.2)	6.83 (14.89)	0.03	2.19

There were a total of 19 AEs of iopamidol that met the positive criteria for the four methods (Table 3). The most frequently reported AEs associated with iopamidol were primarily dyspnoea, with over 200 cases documented. In contrast, the most significant signals were noted for sneezing, throat tightness, and laryngeal oedema. Furthermore, we observed that the AE signals related to yawning (ROR: 12.72, 95% CI: 4.77–33.94) and laryngospasm (ROR: 9.28, 95% CI: 2.99–28.79) were not included in the iopamidol drug label.

**TABLE 3:** Positive signal for iopamidol-related respiratory AEs.

Sort	PT	N	ROR (95%CI)	PRR (X^2^)	IC025	EBGM05
1	Dyspnoea	220	3.44 (3.0,3.93)	3.36 (367.96)	1.54	2.94
2	Sneezing	74	29.43 (23.4,37.02)	29.14 (2003.95)	4.07	23.08
3	Throat tightness	58	18.76 (14.49,24.3)	18.62 (965.13)	3.46	14.34
4	Throat irritation	56	11.13 (8.55,14.48)	11.05 (511.5)	2.85	8.48
5	Respiratory arrest	44	13.12 (9.75,17.65)	13.05 (488.83)	2.93	9.68
6	Pharyngeal oedema	28	14.32 (9.88,20.77)	14.27 (345.04)	2.75	9.83
7	Wheezing	27	4.21 (2.88,6.14)	4.2 (65.77)	1.37	2.87
8	Respiratory distress	21	6.52 (4.25,10.01)	6.51 (97.83)	1.76	4.24
9	Bronchospasm	17	10.08 (6.26,16.22)	10.05 (138.47)	2.06	6.24
10	Laryngeal oedema	15	20.92 (12.59,34.74)	20.87 (283.1)	2.49	12.54
11	Acute respiratory failure	12	5.55 (3.15,9.77)	5.54 (44.6)	1.24	3.14
12	Acute pulmonary oedema	8	12.05 (6.02,24.13)	12.04 (80.88)	1.47	6.01
13	Apnoea	7	7.49 (3.57,15.73)	7.49 (39.32)	1.03	3.56
14	Oropharyngeal discomfort	7	7.37 (3.51,15.47)	7.36 (38.47)	1.01	3.51
15	Pharyngeal swelling	5	5.03 (2.09,12.1)	5.03 (16.14)	0.41	2.09
16	Stridor	5	14.59 (6.06,35.09)	14.58 (63.11)	0.98	6.05
17	Yawning	4	12.72 (4.77,33.94)	12.72 (43.12)	0.63	4.76
18	Oropharyngeal swelling	3	24.42 (7.86,75.86)	24.41 (67.13)	0.39	7.83
19	Laryngospasm	3	9.28 (2.99,28.79)	9.27 (22.12)	0.15	2.98

There were a total of 39 AEs of iopromide that met the positive criteria for the four methods (Table 4). The most frequently reported AEs associated with iopromide included dyspnoea, sneezing, and cough, with more than 250 cases documented. Notably, the most significant signals identified were pharyngolaryngeal discomfort, sneezing, and laryngeal oedema. Additionally, we discovered that certain AE signals related to iopromide, such as sneezing (ROR: 62.29, 95% CI: 56.91–68.17), central cyanosis (ROR: 30.02, 95% CI: 9.61–93.76), pharyngeal hypoaesthesia (ROR: 14.30, 95% CI: 5.93–34.44), and pharyngeal erythema (ROR: 5.73, 95% CI: 2.38–13.79), were not included in the drug labeling.

**TABLE 4:** Positive signal for iopromide-related respiratory AEs.

Sort	PT	N	ROR (95%CI)	PRR (X^2)^	IC025	EBGM05
1	Dyspnoea	948	4.6 (4.31,4.9)	4.45 (2,553.95)	2.05	4.16
2	Sneezing	494	62.29 (56.91,68.17)	60.99 (28,429.85)	5.6	54.36
3	Cough	270	2.63 (2.33,2.96)	2.61 (268.25)	1.2	2.31
4	Nasal congestion	246	11.67 (10.29,13.23)	11.56 (2,362.57)	3.28	10.14
5	Throat irritation	205	12.56 (10.94,14.42)	12.46 (2,150.91)	3.35	10.8
6	Throat tightness	185	18.46 (15.96,21.34)	18.32 (3,007.4)	3.84	15.73
7	Laryngeal oedema	120	52.49 (43.79,62.93)	52.22 (5,900.46)	4.91	42.65
8	Dysphonia	109	5.0 (4.14,6.03)	4.98 (345.94)	1.99	4.12
9	Pharyngeal oedema	85	13.39 (10.81,16.58)	13.34 (965.51)	3.23	10.72
10	Wheezing	85	4.07 (3.29,5.04)	4.06 (195.79)	1.66	3.28
11	Respiratory arrest	84	7.68 (6.2,9.52)	7.65 (484.62)	2.51	6.16
12	Bronchospasm	69	12.61 (9.95,15.98)	12.57 (731.29)	3.08	9.87
13	Tachypnoea	63	12.58 (9.82,16.12)	12.55 (666.48)	3.04	9.75
14	Respiratory distress	61	5.82 (4.53,7.49)	5.81 (242.35)	2.06	4.51
15	Oropharyngeal discomfort	58	18.9 (14.59,24.48)	18.85 (973.03)	3.47	14.45
16	Asphyxia	40	12.9 (9.45,17.61)	12.88 (436.06)	2.86	9.39
17	Suffocation feeling	24	25.56 (17.09,38.23)	25.54 (559.88)	3.10	16.90
18	Acute pulmonary oedema	22	10.2 (6.71,15.5)	10.19 (181.56)	2.26	6.68
19	Nasal obstruction	22	38.11 (25.01,58.08)	38.08 (781.76)	3.25	24.60
20	Choking sensation	21	10.22 (6.66,15.69)	10.21 (173.79)	2.23	6.63
21	Oropharyngeal swelling	19	48.28 (30.65,76.04)	48.24 (861.48)	3.18	30.03
22	Laryngospasm	18	17.19 (10.81,27.33)	17.17 (272.23)	2.54	10.73
23	Nasal discomfort	17	5.54 (3.44,8.92)	5.54 (63.03)	1.46	3.43
24	Nasal oedema	15	23.35 (14.04,38.83)	23.33 (317.52)	2.55	13.90
25	Apnoea	14	4.6 (2.72,7.78)	4.6 (39.37)	1.14	2.72
26	Stridor	14	12.57 (7.44,21.26)	12.57 (148.28)	2.08	7.40
27	Laryngeal discomfort	10	43.71 (23.38,81.72)	43.7 (409.68)	2.28	22.96
28	Pharyngolaryngeal discomfort	10	256.18 (133.42,491.9)	256.07 (2,294.06)	2.48	120.46
29	Nasal pruritus	8	17.96 (8.96,36.02)	17.96 (127.14)	1.67	8.89
30	Upper airway obstruction	8	23.56 (11.74,47.27)	23.55 (171.05)	1.78	11.63
31	Respiratory tract oedema	7	27.07 (12.85,57.03)	27.06 (173.72)	1.64	12.71
32	Upper respiratory tract congestion	6	5.24 (2.35,11.68)	5.24 (20.54)	0.61	2.35
33	Laryngeal stenosis	5	26.7 (11.06,64.48)	26.7 (122.31)	1.15	10.94
34	Pharyngeal erythema	5	5.73 (2.38,13.79)	5.73 (19.48)	0.50	2.38
35	Pharyngeal hypoaesthesia	5	14.3 (5.93,34.44)	14.29 (61.45)	0.97	5.90
36	Mouth breathing	3	18.75 (6.02,58.41)	18.75 (50.02)	0.33	5.98
37	Cyanosis central	3	30.02 (9.61,93.76)	30.02 (83.11)	0.41	9.50
38	Diffuse alveolar damage	3	6.67 (2.15,20.7)	6.66 (14.4)	0.02	2.14
39	Use of accessory respiratory muscles	3	21.02 (6.74,65.49)	21.01 (56.68)	0.35	6.69

There were a total of 30 AEs of iopromide that met the positive criteria for the four methods (Table 5). The most frequently reported AEs associated with ioversol included dyspnoea, throat irritation, and sneezing, each exceeding 200 cases. The most pronounced signals were noted for pharyngolaryngeal discomfort, sneezing, and laryngeal discomfort. Furthermore, pharyngeal hypoaesthesia (ROR: 49.05, 95% CI: 23.30–103.24), yawning (ROR: 11.93, 95% CI: 4.96–28.69), nasal congestion (ROR: 7.48, 95% CI: 5.86–9.55), and dry throat (ROR: 4.49, 95% CI: 2.14–9.43) are notable AE signals that were not included in the drug labeling for ioversol.

**TABLE 5:** Positive signal for ioversol-related respiratory AEs.

Sort	PT	N	ROR (95%CI)	PRR (X^2)^	IC025	EBGM05
1	Dyspnoea	439	5.22 (4.75,5.75)	5.03 (1,429.17)	2.18	4.57
2	Throat irritation	212	32.18 (28.08,36.89)	31.49 (6,229.03)	4.58	27.33
3	Sneezing	207	62.87 (54.74,72.21)	61.53 (12,200.68)	5.36	53.02
4	Cough	180	4.31 (3.72,4.99)	4.24 (448.06)	1.84	3.66
5	Throat tightness	140	34.3 (29.01,40.55)	33.81 (4,433.83)	4.53	28.44
6	Pharyngeal oedema	66	25.46 (19.97,32.45)	25.29 (1,533.24)	3.85	19.76
7	Wheezing	65	7.63 (5.97,9.73)	7.58 (371.19)	2.43	5.93
8	Nasal congestion	65	7.48 (5.86,9.55)	7.43 (361.8)	2.4	5.82
9	Bronchospasm	56	25.05 (19.25,32.59)	24.91 (1,279.85)	3.74	19.06
10	Laryngeal oedema	52	54.91 (41.76,72.21)	54.62 (2,711.87)	4.36	41.16
11	Dysphonia	41	4.58 (3.37,6.23)	4.57 (114.27)	1.63	3.36
12	Respiratory distress	36	8.4 (6.05,11.65)	8.37 (233.31)	2.33	6.02
13	Respiratory arrest	36	8.03 (5.79,11.14)	8 (220.33)	2.27	5.76
14	Oropharyngeal discomfort	17	13.45 (8.35,21.66)	13.43 (195.11)	2.31	8.32
15	Pharyngeal swelling	11	8.31 (4.6,15.02)	8.31 (70.59)	1.53	4.59
16	Nasal discomfort	10	7.96 (4.28,14.8)	7.95 (60.66)	1.41	4.27
17	Apnoea	8	6.42 (3.21,12.85)	6.42 (36.55)	1.04	3.20
18	Laryngeal discomfort	8	85.17 (42.37,171.23)	85.1 (655.33)	2.07	41.73
19	dry throat	7	4.49 (2.14,9.43)	4.49 (18.99)	0.62	2.14
20	Pharyngeal hypoaesthesia	7	49.05 (23.3,103.24)	49.02 (326.51)	1.78	23.10
21	Laryngospasm	6	13.93 (6.25,31.04)	13.92 (71.79)	1.20	6.23
22	Acute pulmonary oedema	6	6.77 (3.04,15.09)	6.77 (29.47)	0.80	3.04
23	Yawning	5	11.93 (4.96,28.69)	11.92 (49.94)	0.90	4.95
24	Hyperventilation	5	5.44 (2.26,13.08)	5.44 (18.1)	0.46	2.26
25	Upper respiratory tract congestion	3	6.4 (2.06,19.85)	6.39 (13.64)	0.01	2.06
26	Stridor	3	6.55 (2.11,20.34)	6.55 (14.1)	0.01	2.11
27	Suffocation feeling	3	7.73 (2.49,23.99)	7.73 (17.55)	0.08	2.49
28	Respiratory tract oedema	3	28.16 (9.06,87.58)	28.16 (78.2)	0.41	9.01
29	Pharyngeal paraesthesia	3	13.94 (4.49,43.28)	13.93 (35.93)	0.27	4.48
30	Pharyngolaryngeal discomfort	3	174.62 (55.37,550.73)	174.56 (502.63)	0.50	53.75

### Comparison of significant safety signals among iohexol, iopamidol, iopromide, and ioversol AEs

**FIGURE 3:**
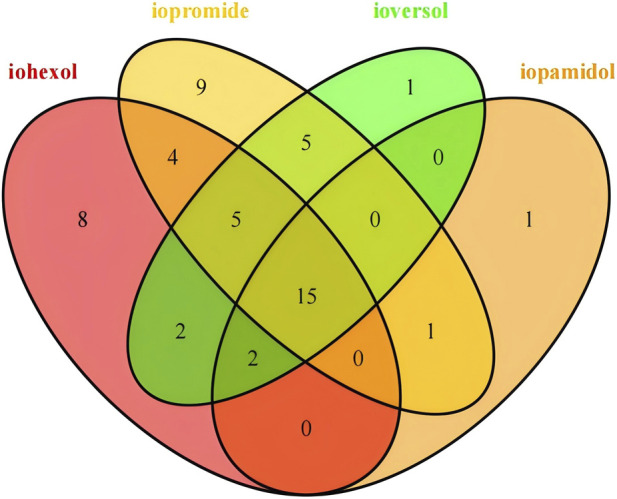
Comparison of safety signals for AEs related to the respiratory among four ICM Drugs. Illustrates the comparison of AEs associated with the target drug in the respiratory system, primarily highlighting the interrelationships among the specific safety signals detected for the four drugs.

**FIGURE 4:**
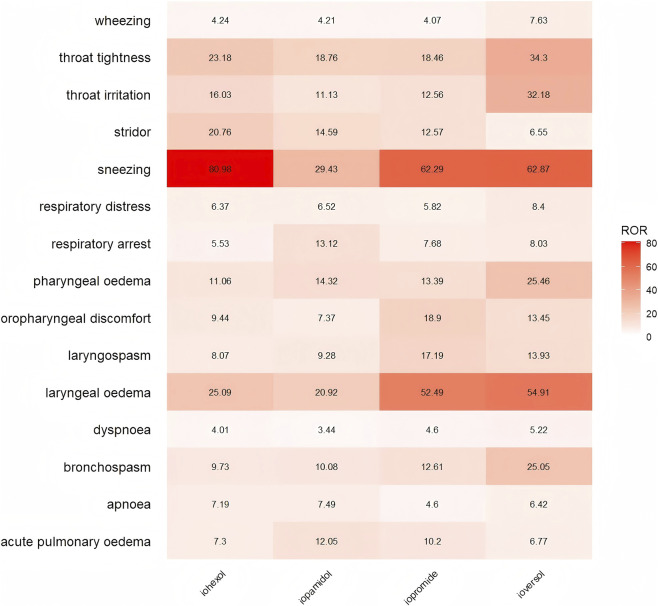
Comparison of the signal strength of AEs shared by four ICM Drugs. Presents the research findings derived from Figure 3, which compares the signal strengths of 15 shared respiratory AEs among the four drugs.

Through Venn analysis, we identified 15 AEs related to the respiratory system that are common among the four ICM drugs (Figure 3). Additionally, we observed that iopamidol and ioversol each have one specific AE, whereas iohexol has eight and iopromide has nine. When analyzing the signal strength of the 15 common AEs (using the ROR as an example), we found that both sneezing and laryngeal oedema exhibited extremely high signal strength among the four ICM drugs (Figure 4).

### Gender subgroup analysis

We conducted a gender subgroup analysis for ICM-related respiratory AEs. Our findings indicate that females (ROR: 4.26, 95% CI: 4.09–4.43) have a higher risk of developing ICM-related respiratory AEs compared to males (ROR: 2.88, 95% CI: 2.73–3.03). In the gender subgroup analysis, the most common ICM-related respiratory AEs for both males and females included dyspnea, sneezing, cough, throat irritation, and throat tightness. Further comparison of signal intensities revealed that females exhibited higher signals than males for dyspnea, with ROR values of 4.53 versus 3.43, respectively. Sneezing events showed the strongest signal in males, with an ROR reaching 64.43 compared to 55.96 in females. For cough, females had an ROR of 3.35, which is slightly higher than the male value of 2.34. Throat irritation demonstrated an ROR of 16.69 in females, also exceeding the male value of 12.47. Throat tightness displayed similar ROR values between genders (20.43 for males vs. 19.04 for females). Overall, females generally exhibited stronger signals of respiratory hypersensitivity-like reactions to ICM.

## Discussion

This study utilized a large, real-world AE reporting database to evaluate the drug safety profiles of iohexol, iopamidol, iopromide, and ioversol. A systematic analysis employing four statistical methods was conducted, through which we identified common AEs related to the respiratory system, as well as AEs not documented in the product instructions. However, it is essential to emphasize that the present study, which utilizes the FAERS database for disproportionality analysis, primarily yields the metric of “signal intensity.” These signals represent relative reporting frequencies rather than the actual incidence or absolute risk of adverse reactions. The magnitude of these signals is substantially influenced by non-safety factors such as drug utilization volume, clinical awareness, medical practices, and reporting preferences. Consequently, our analysis focuses on the relative signal characteristics of the four ICMs within the existing reporting framework, thereby generating scientific hypotheses that warrant validation through prospective studies.

The traditional view posits that the relatively low osmolality of non-ionic ICMs significantly reduces respiratory risks compared to ionic formulations, which have osmolalities exceeding 1,500 mOsm/kg. The number of reports regarding systemic AEs associated with ICM in the FAERS database is insufficient for quantitative comparative analysis (diatrizoate meglumine, n = 20; ioxaglate, n = 189). Nevertheless, prospective studies have confirmed the safety advantages of non-ionic ICMs. Wolf et al. conducted a multicenter study comparing the adverse reactions of ionic and non-ionic ICM in clinical practice. Their findings indicated that the incidence of adverse reactions in the ionic ICM group was significantly higher than that in the non-ionic ICM group (4.17% vs. 0.69%, p < 0.001), with the reactions in the ionic group being more severe (p < 0.005) (Wolf et al., 1989). This disparity is primarily attributed to the higher osmotic pressure of ionic ICM, which is approximately 6–8 times that of plasma osmotic pressure. Such elevated osmotic pressure results in greater irritability of vascular endothelial cells and increased chemical toxicity, thereby raising the occurrence of AEs (Zeng et al., 2024; Siegle, 1993). However, this study suggests that adverse respiratory reactions remain non-negligible even among non-ionic ICM, as evidenced by the signal strengths of all four ICM being greater than 1. Although the osmolality of non-ionic ICM is lower than that of ionic ICM, it still significantly exceeds plasma osmolality, which ranges from 280 to 310 mOsm/kg. For instance, the osmolality of iohexol is 830 mOsm/kg, approximately 2.8 times that of plasma. This elevated osmolality may result in increased pulmonary blood flow and mast cell degranulation, leading to the release of histamine, interleukins, and leukotrienes, which can induce bronchial smooth muscle contraction (Grandinetti et al., 2024). Furthermore, in clinical practice, confounding factors such as the patient’s baseline risk (e.g., a history of asthma or the use of β-blockers) and the rate of administration may exacerbate these respiratory risks associated with non-ionic ICM.

In this study, we found that females accounted for a significantly higher proportion of AE reports related to the respiratory system associated with ICM, at 63.47%. Following this, we further analyzed the gender ratio of systemic AEs (after excluding missing values), where females represented 64.61% (n = 5,425). This is consistent with the results of the gender subgroup analysis we conducted. This phenomenon aligns with the observations made regarding respiratory system AEs. The higher reporting rate among females may reflect a reporting bias or demographic differences in the utilization of imaging. Estrogen may enhance mast cells’ ability to release histamine, exacerbating airway hyperresponsiveness and predisposing individuals to a range of adverse respiratory reactions (Radzikowska and Golebski, 2023). Females are more susceptible to autoimmune diseases, such as systemic lupus erythematosus, which may indirectly elevate the risk of respiratory complications (Mousavi et al., 2020). Conversely, the female immune system may exacerbate the IL-17A-mediated airway inflammatory response induced by ICM, although the specific mechanisms remain unclear at present (Fuseini and Newcomb, 2017). It is speculated that this could be related to the regulatory role of genes associated with sex chromosomes. The age distribution of patients receiving ICM predominantly falls within the 45–65 age group. This is primarily due to the fact that patients in this age range are more prone to high-incidence diseases such as cardiovascular diseases, diabetes, and renal insufficiency, which necessitate frequent imaging examinations. Additionally, comorbidities may exacerbate responses within the respiratory system. The reports submitted to the national registry are primarily from the United States, with the submitters mainly comprising pharmacists and physicians. This trend is largely attributable to the United States’ position as one of the top global users of ICM, its strong emphasis on pharmacovigilance, and a corresponding high level of awareness among clinicians and pharmacists regarding the importance of reporting. The respiratory adverse reactions associated with the four drugs predominantly occurred within 0–30 days after administration, a phenomenon primarily linked to the metabolic characteristics of the drugs and the immune responses involved. Acute adverse reactions, such as bronchospasm and laryngeal oedema, typically manifest within 30 min following the injection of ICM and are predominantly linked to IgE-mediated immediate hypersensitivity reactions. Certain adverse effects may arise from Th2-type immune activation or the activation of the complement alternative pathway, generally occurring within 3–7 days post-medication administration, such as respiratory failure associated with iopamidol (Khan et al., 2015). A minority of patients may experience related adverse reactions approximately 1 month later, such as diffuse alveolar damage induced by iopromide, potentially linked to chronic inflammation triggered by repeated exposure or drug accumulation. The resulting outcomes predominantly involve hospitalization, including respiratory tract oedema induced by iohexol (ROR: 32.86, 95% CI: 15.60–69.22), acute pulmonary oedema caused by iopamidol (ROR: 12.05, 95% CI: 6.02–24.13), asphyxia triggered by iopromide (ROR: 12.90, 95% CI: 9.45–17.61), and bronchospasm induced by ioversol (ROR: 25.05, 95% CI: 19.25–32.59). These severe adverse reactions, characterized by high signal detection, are often inadequately managed through outpatient treatment alone. Since 2014, the use of iohexol has exhibited a significant upward trend, likely due to its advantages in imaging clarity, which has facilitated its broader application in cardiovascular and neurointerventional procedures, thereby contributing to an increase in reported AEs. Additionally, we must consider the potential impact of the market usage patterns and reporting ecosystems of the four ICMs. The number of reports for iopromide and iohexol is significantly higher than that for iopamidol. This discrepancy may be partially attributed to their earlier market launch, particularly in the U.S. market, which serves as the primary source of research data, as well as their broader global usage base. Conversely, clinicians’ awareness of specific adverse drug reactions, such as allergic reactions associated with iopromide, may also contribute to reporting bias. These factors prevent us from equating the quantity of reports or the ranking of signal strength with intrinsic safety levels. The more significant value of this study lies in revealing that even among equally safe non-ionic ICMs, there exists heterogeneity in their adverse reaction reporting profiles, indicating the necessity for further population-based epidemiological research.

Through data analysis, we found that all four drugs exhibited extremely high sneezing signal intensity. However, it is noteworthy that the package inserts of iohexol and iopromide do not mention adverse reactions related to sneezing. Sneezing is a reflexive protective action mediated by the trigeminal nerve when the upper respiratory tract is stimulated by external irritants such as dust or pollen (Jiang et al., 2024). Currently, there exists a gap in understanding the mechanisms by which ICM directly induces sneezing. Based on a comprehensive review of the relevant literature, we propose the following mechanistic hypothesis regarding this adverse effect. ICM can directly stimulate mast cells in the nasal mucosa, prompting the rapid release of pre-stored histamine granules (Kun and Jakubowski, 2012). The released histamine binds to H1 receptors on the trigeminal nerve endings, activating transient receptor potential vanilloid receptor-1 (TRPV1) channels (Shim et al., 2007). Neural signals are transmitted via the afferent fibers of the trigeminal nerve to the olfactory bulb and medulla oblongata (Li et al., 2021). After central integration, the respiratory muscles are activated, resulting in the sneeze reflex. This reaction is not dependent on IgE mediation and is classified as an anaphylactoid reaction. ICM typically possesses a certain level of osmotic pressure (>600 mOsm/kg), which may lead to dehydration and shrinkage of nasal mucosal epithelial cells, disrupting tight junctions between cells and allowing ICM to directly contact exposed nerve endings. The iodine molecules in ICM can also penetrate the mucus layer and covalently bind to TRPA1 channels on the trigeminal nerve endings, triggering conformational changes that lead to the influx of calcium ions (Su et al., 2016). On one hand, calcium influx triggers the rapid fusion of synaptic vesicles through presynaptic protein mechanisms, promoting the retrograde release of substance P into surrounding tissues (Bose et al., 2024). After substance P binds to the NK1 receptor, it activates mast cell degranulation and increases vascular permeability, triggering local plasma extravasation, mast cell chemotaxis, and other neurogenic inflammatory responses (Kun and Jakubowski, 2012; Sumpter et al., 2015). On the other hand, calcium influx initiates the action potential of the trigeminal nerve, which is transmitted to the brainstem via Neuromedin B (NMB), completing the sneeze reflex through the pontomedullary circuit (Li et al., 2021). Moreover, sneezing, as a conspicuous symptom, is more easily recognized and recorded by both patients and healthcare professionals, leading to a higher reporting rate compared to less noticeable reactions, such as diffuse alveolar damage. Some patients suffer from allergic rhinitis, and the use of ICM may amplify the sneeze reflex. Some patients routinely use β-blockers or nonsteroidal anti-inflammatory drugs (NSAIDs), which may inhibit anti-inflammatory pathways and indirectly enhance the sneeze response. Therefore, for certain high-risk groups (such as individuals with allergic constitutions and patients with allergic rhinitis), it is recommended to prioritize the use of low-osmolarity ICM (e.g., iopamidol) and to prophylactically administer antihistamines before conducting contrast examinations. Meanwhile, the observation period should be extended, and patients should be closely monitored for any exacerbation of sneezing symptoms, such as progression to laryngeal oedema or bronchospasm. Regarding the sneeze reflex issue induced by ICM mentioned above, future research could further clarify the direct effects of different ICM on trigeminal nerve activity through *in vitro* experiments, as well as the proportion of IgE-mediated and non-IgE-mediated pathways.

We also found that all four ICM exhibited extremely high signal strength for laryngeal oedema in adverse respiratory reactions. Laryngeal oedema is one of the most perilous complications associated with ICM-related allergic reactions, as it can rapidly progress to airway obstruction or even fatal asphyxiation. In clinical practice, the mechanism by which ICM causes laryngeal edema remains unclear. However, we surmise that the mechanism by which ICM induces laryngeal edema is analogous to the physiological processes that trigger sneezing. ICM can facilitate the influx of calcium ions into cells through their specific osmotic pressure effect, thereby triggering the release of substance P and inducing neurogenic inflammatory exudation (Su et al., 2016). ICM can also directly activate mast cells via the MRGPRX2 receptor, resulting in the release of mediators such as histamine and leukotrienes, which increases capillary permeability (Navinés-Ferrer et al., 2018). Furthermore, the iodobenzene ring structure of ICM displays a similar electron cloud distribution to the active center of angiotensin-converting enzyme (ACE), particularly in the substrate angiotensin I binding domain (Acharya et al., 2024). This similarity reduces enzyme-substrate binding efficiency due to steric hindrance. Notably, in patients concurrently using ACE inhibitors, undergoing ICM examination may lead to a significant increase in plasma bradykinin concentration (Tom et al., 2002). Excessive bradykinin induces capillary leakage through B2 receptor activation and upregulates TRPV1 channels, exacerbating tissue oedema (Yin et al., 2021). Therefore, patients with arteriosclerosis or those who frequently use ACE inhibitors may have an increased risk of laryngeal oedema via the bradykinin pathway. This patient demographic typically includes middle-aged and elderly individuals (45–65 years old), which aligns with the results obtained from our analysis of basic information data. Laryngeal oedema, recognized as an emergency condition, is readily identified and reported by physicians. In contrast, mild manifestations of laryngeal oedema, such as hoarseness, may often be overlooked. In certain instances, this condition may be misdiagnosed as adverse reactions associated with other systems, leading to laryngeal oedema not being documented separately. For example, in the statistical analysis of iopamidol, the records of anaphylactic shock (ROR: 52.78, 95% CI: 44.83–62.15) may obscure the true risk of laryngeal oedema, resulting in a discrepancy between the perceived signal strength of laryngeal edema and its actual risk. The FAERS database provides only preliminary information regarding the occurrence of laryngeal oedema and lacks a severity grading system. It is also deficient in critical parameters such as drug concentration and injection speed, which precludes an in-depth analysis of the dose-response relationship between these factors.

Through an in-depth analysis of the Venn diagram, we found that, compared to iohexol and iopromide, iopamidol is associated with a unique AE—acute respiratory failure (ROR: 5.55, 95% CI: 3.15–9.77). Similarly, ioversol exhibits a distinct AE—hyperventilation (ROR: 5.44, 95% CI: 2.26–13.08). However, we maintain a skeptical stance regarding these analytical results. As non-ionic ICMs, iohexol, iopamidol, iopromide, and ioversol exhibit highly similar physicochemical properties (e.g., osmotic pressure around 600–800 mOsm/kg), with adverse reaction rates typically varying only between 0.77% and 1.74% (Suh et al., 2019). Acute respiratory failure is linked to IgE-mediated allergic reactions, and clinical studies indicate that the cross-reactivity rate among ICMs can be as high as 30% (Schmid et al., 2021). For the AEs specific to a single formulation, further confirmation through toxicological studies is necessary.

Based on the analysis results of this study, we have clarified the respiratory risk spectrum associated with common non-ionic ICM and facilitated the transition from an overarching focus on “overall safety” to a more nuanced approach of “precise prevention and control.” Clinically, it is imperative to implement preventive measures to mitigate the occurrence of high-signal AEs. For example, administering oral prednisone at a dosage of 40–60 mg 12 h and 2 h prior to contrast imaging, in conjunction with antihistamines such as cetirizine (10 mg), has been shown to effectively reduce the risk of laryngeal oedema. Furthermore, it is essential to remain vigilant regarding potential AEs that may not be explicitly outlined in the drug’s prescribing information.

## Limitations

First, as a study based on a spontaneous reporting system, a key structural limitation that this research cannot overcome is the absence of exposure denominator information. Signal detection methods, such as ROR and PRR, partially mitigate this limitation by comparing proportions rather than absolute numbers; however, the results may still be influenced by residual confounding due to variations in the prescription population across different drugs. Second, the FAERS data is susceptible to reporting bias, confounding bias, and uneven quality of reported information, which may compromise the integrity of the detected signals. Therefore, as a retrospective pharmacovigilance study, this research reveals only a statistical association between the drug and AEs, rather than a causal relationship. Thirdly, the mechanisms proposed in this paper, such as TRPV1 activation and the bradykinin pathway, are speculative hypotheses based on literature. The FAERS data itself cannot provide direct evidence at the molecular or pathway level, and these hypotheses require further validation through experimental research in the future. Furthermore, given that ICM is primarily utilized as an acute agent for diagnostic imaging rather than as a continuous medication treatment, we analyzed the occurrence time and median occurrence time of AEs during the research process. However, it was observed that most reports did not specify the exact occurrence time in their analyses. Consequently, it is imperative to clearly articulate that the direct temporal association between ICM and the reported events is a statistical inference and does not establish a causal relationship.

## Conclusion

This study presented the first comprehensive analysis of AEs triggered by four common nonionic ICM within the respiratory system, utilizing multidimensional signal mining techniques. We identified 15 respiratory-related AEs that are common among the four ICMs. Notably, sneezing and laryngeal edema exhibited exceptionally high signal intensities, warranting significant clinical attention. These findings provided critical safety references for clinical drug administration. Furthermore, the causal relationship between these drugs and the observed AEs requires further verification through experimental studies in the future.

## Data Availability

The original contributions presented in the study are included in the article/Supplementary Material, further inquiries can be directed to the corresponding authors.
