# Supplementary material for: Pharmacovigilance analysis of iodinated contrast media related respiratory adverse effects based on the FDA adverse event reporting system
Source: Front Pharmacol. 2026 Mar 16;17:1737135. doi: 10.3389/fphar.2026.1737135 (PMC13033718; doi:10.3389/fphar.2026.1737135)
Supplement: Supplementary file 1 [file Table1.docx]

**Supplementary Table S1. Four fold table of disproportionality measures**

| **Type of drug** | **targeted adverse event** | **other adverse events** | **Total** |
| --- | --- | --- | --- |
| Target drug | a | b | a+b |
| Other drugs | c | d | c+d |
| Total | a+c | b+d | a+b+c+d |
