# Supplementary material for: Pharmacovigilance analysis of iodinated contrast media related respiratory adverse effects based on the FDA adverse event reporting system
Source: Front Pharmacol. 2026 Mar 16;17:1737135. doi: 10.3389/fphar.2026.1737135 (PMC13033718; doi:10.3389/fphar.2026.1737135)
Supplement: Supplementary file 3 [file Table3.docx]

**Supplementary Table S3. Median time to onset of significant PT for four drugs**

| **Sort** | **PT** | **N** | **Median（day）** |
| --- | --- | --- | --- |
| **iohexol** | | | |
| 1 | dyspnoea | 13 | 1 |
| 2 | sneezing | 1 | 2 |
| 3 | cough | 1 | 5 |
| 4 | throat irritation | unknown | unknown |
| 5 | throat tightness | 1 | 1 |
| 6 | nasal congestion | 1 | 2 |
| 7 | dysphonia | unknown | unknown |
| 8 | wheezing | unknown | unknown |
| 9 | pharyngeal oedema | 2 | 6 |
| 10 | respiratory distress | 2 | 3 |
| **iopamidol** | | | |
| 1 | dyspnoea | 5 | 2 |
| 2 | sneezing | 1 | 60 |
| 3 | throat tightness | unknown | unknown |
| 4 | throat irritation | unknown | unknown |
| 5 | respiratory arrest | 1 | 3 |
| 6 | pharyngeal oedema | 2 | 1 |
| 7 | wheezing | unknown | unknown |
| 8 | respiratory distress | 1 | 1 |
| 9 | bronchospasm | unknown | unknown |
| 10 | laryngeal oedema | unknown | unknown |
| **iopromide** | | | |
| 1 | dyspnoea | 12 | 5.5 |
| 2 | sneezing | 1 | 59 |
| 3 | cough | 1 | 31 |
| 4 | nasal congestion | 1 | 59 |
| 5 | throat irritation | unknown | unknown |
| 6 | throat tightness | 1 | 17 |
| 7 | laryngeal oedema | 1 | 12 |
| 8 | dysphonia | 1 | 1 |
| 9 | pharyngeal oedema | 1 | 1 |
| 10 | wheezing | unknown | unknown |
| **ioversol** | | | |
| 1 | dyspnoea | 3 | 3 |
| 2 | throat irritation | unknown | unknown |
| 3 | sneezing | 1 | 2 |
| 4 | cough | unknown | unknown |
| 5 | throat tightness | 1 | 1 |
| 6 | pharyngeal oedema | unknown | unknown |
| 7 | wheezing | unknown | unknown |
| 8 | nasal congestion | unknown | unknown |
| 9 | bronchospasm | unknown | unknown |
| 10 | laryngeal oedema | unknown | unknown |

N, refers to the number of instances where this PT documents the time of occurrence.
